# Study on mange mite of camel in Raya-Azebo district, northern Ethiopia

**Published:** 2014

**Authors:** Nesibu Awol, Semere Kiros, Yisehak Tsegaye, Mohammed Ali, Birhanu Hadush

**Affiliations:** 1 *Department of Veterinary Medicine, College of Veterinary Medicine, Mekelle University, Tigray, Ethiopia; *; 2 *Raya Azebo District Agricultural Office, Tigray, Ethiopia;*; 3 *Department of Veterinary Pathology and Parasitology, School of Veterinary medicine, Welayta Sodo University,* *Wolaita Sodo, Ethiopia.*

**Keywords:** Camel, Mange mite, Raya-Azebo, *Sarcoptes *

## Abstract

A cross-sectional study was carried out to determine the prevalence and species of camel mange mite infestation in Raya-Azebo district, Northern part of Ethiopia. Accordingly, Three hundred and eighty-four camels were examined and mange mite infestation was detected on 64 of camels. Only *Sarcoptes scabiei *var*. cameli* was identified as the only mite species in all skin scraping samples collected from the suspected mange mite lesions. There was significant difference in the prevalence of mange mite infestation between male and female camels (*p* < 0.05) but no significance difference was observed among the age groups and body condition score of camels (*p* > 0.05). The result indicated that camel mange mite infestation was a problem in northern part of Ethiopia, hence, further studies and strategic control measures are recommended to reduce the effect of mange mite infestation on camel husbandry.

## Introduction

Camel (*Camelus dromedarius*) is an important domestic animal species uniquely adapted to the hot and arid environment. In Ethiopia camels are found in northeastern, eastern, southeastern and southern parts of the country.^1^ According to the animal population census, the camel population in Ethiopia is estimated to be 1.70 million.^2^ The major ethnic groups owning camels in Ethiopia are the Beja, Afar, Somali, and Borana.^3^

Despite their huge socio-economic importance and adaptation in hot and arid environment, camels are still affected by various diseases. The most important diseases are sarcoptic mange, caused by *Sarcoptes* *scabiei* var. *cameli*, and trypanosomosis (surra), due to *T. evansi*.^4^^-^^7^ Camel mange mite infestation was also indicated as the second most important camel disease after trypanosomiasis (surra) in terms of its effect on production in camel herds across the world.^8^^,^^10^ Sarcoptic mange is also of zoonotic nature.^7^ Camel owners are the main sufferers due to close association with camels.^7^

Camel mange is an extremely contagious skin disease, characterized by scab formation, pruritic dermatitis, thickening and corrugation of skin and hair loss, and caused by the parasitic mite.^8^ Sarcoptic and chorioptic mange mites have been reported from camel. However, sarcoptic mange caused by *Sarcoptes scabiei *var.* cameli* is the most common, extremely contagious and serious problem in camels.^7^^,^^9^^,^^11^ Chorrioptic mange is mild and occurs only rarely in camel.^11^

Numerous studies have been conducted on camel mange mite infestation worldwide and a prevalence ranging from 3.54% to 83.00% have been recorded by various investigators.^12^^-^^19^ In eastern Ethiopia camel mange mite infestation was reported at a prevalence of 10.68%,^17^ 27.80%,^19^ and 32.20%.^12^ However, there is no available data regarding the prevalence of this disease in the northern part of the country. The present study was therefore conducted to collect data on camel mange mite infestation in Raya-Azebo district, northern part of Ethiopia. 

## Materials and Methods

**Study area** **. **The study was conducted in Raya-Azebo district, Tigray Region, northern Ethiopia from May, 2011- July, 2011. Raya-Azebo district is located at latitude of 12° - 180° north and longitude of 38° - 39° east. The average elevation of the district is 1470 -2370 meter above sea level. The mean annual precipitation is 610.50 (351-870) mm. The mean minimum and maximum annual temperature for the area are 15 ˚C and 30 ˚C, respectively. Raya Azebo has three agro climatic zones of which 47.00%, 50.00% and 3.00% are lowland, midland and high land, respectively.^20^

**Study type** **and study animals** **.** The study was a cross-sectional study undertaken to establish the prevalence and to identify the species of mange mite infestation in camel. The study was conducted on 384 camels selected by simple random sampling method without discrimination of their age, sex and body condition. 

**Sample size determination** **. **The sample size was determined following the formula described by Thrusfield *et al.*^21^ By considering the expected prevalence of 50.00% and 5.00% absolute precision with 95.00% confidence level the sample size was found to be 384 camels.^22^

**Data collection** **. **General physical examination was conducted on each camel. The distribution of mange mite lesion on the animal body was carefully examined and recorded. All data regarding the age, sex, body condition and other related information of the animals were recorded appropriately. The age and body condition of camels were determined based on their dentition and hump structure.^23^^,^^24^

**Sample collection and identification of** **mite.** Skin scraping samples from 64 suspected cases of mange mite infection were collected for mange mite examination using scalpel blade by scraping the edges of the lesions until capillary bleeding was seen and preserved in 10% formalin and identification of mite were carried out according to the standard technique recommended before.^24^^-^^26^

**Data analysis. **The data were analyzed using SPSS (Version 17; SPSS Inc., Chicago, USA) software. The Chi-square (*X*^2^) test was used to assess differences in the prevalence of the infection among, body condition score, sex and age groups. In all cases, 95% confidence intervals and *p* < 0.05 were set for significance.

## Results

Out of the 384 camels examined, 64(16.70%) of camels had mange mite infestation. Lesions of mange mite infestation were observed most commonly on the face (72.75%), neck region (58.30%), abdominal region (47.30%), inner surface of the thighs (32.10%) and inguinal region (29.50%) of infected camels. Clinically, the suspected lesions of mange mite were characterized by hair loss, scab formation, thickening and corrugation of skin and intense itching was also observed on some camels during the study period. Only *Sarcoptes scabiei *var.* cameli* was identified as the only mite species in all skin scraping samples (n = 64) collected from the suspected lesions. There was significant different in the prevalence of mange mite infestation between male and female camels (*p* < 0.05); with a prevalence of 24.20% in female and 14.20% in male camels but no significance difference was observed among the age groups and body condition score of camels (*p* > 0.05), (Table 1).

**Table 1:** The prevalence of mange mite infestation based on the age, sex and body condition score of camels in Raya-Azebo district, northern Ethiopia.

**Risk factors **	**No. of camels Examined**	**No. of positive camels (%)**	***X*** ^2 ^ **value**	***p –*** *** value***
**Age (year)**	384	64 (16.70%)	6.02	0.19
**1-4**	70	17(24.30%)		
**4-8**	80	9(11.20%)		
**8-12**	85	17(20. 00%)		
**12-16**	76	11(14. 50%)		
**>** **16**	73	10(13.70%)		
**Sex (Total)**	384	64 (16.70%)	5.17	0.02
**Female **	95	23(24.20%)		
**Male **	289	41(14.20%)		
**Body condition score (Total) **384		64(16.70%)	0.55	0.76
**Thin **	1	0(0)		
**Moderate **	234	37(15.80%)		
**Good **	149	27(18.10%)		

## Discussion

An overall prevalence of 16.70% of mange mite infestation was recorded during this study which was higher than the prevalence of 3.54% recorded from Nigeria.^18^ However, this result was almost comparable with the prevalence of 10.68% in eastern Ethiopia,^17^ 13.40% in Pakistan^15^ but lower than the prevalence of 27.80% and 32.20% reported from eastern Ethiopia,^12^^,^^19^ 31.50% from Borana, south Ethiopia,^16^ 83.00% from Jordan^14^ and 31.60% from Sudan.^13^ This variation in the prevalence of camel mange mite might be attributed to the different management systems and environmental condition that exist among those areas. 

There was significant difference in the prevalence of mange mite infestation between male and female camels (*p* < 0.05), however, no significant variation (*p* > 0.05) was observed on the basis of their age groups and body condition score of camels. Higher prevalence of mange mite infestation in female camels may be associated with some hormonal influences. The higher level of prolactin and progesterone hormones could make the females more susceptible to any infection.^27^ Additionally, pregnancy and lactation stress could also aggravate the susceptibility of the female camels to infection. The slight increase in the prevalence of mite infestation in camels with the age of 1-4 years than the rest age groups could be due to the undeveloped acquired immunity of young animals.^17^ In this study *Sarcoptes scabiei *var.* cameli* was identified as the only mite species from all collected samples of skin scrapings. This observation is in a general agreement with various authers^2^^,^^3^^,^^6^^,^^12^^,^^15^ Even though both sarcoptic and chorioptic mange mites have been reported, sarcoptic mange caused by *Sarcoptes scabiei *var.* cameli* is the most common, extremely contagious and serious problem in camels.^7^^,^^9^^,^^10^

Lesions of mange mite infestation were observed most commonly on the head, neck, abdominal regions, inner surface of the thighs and inguinal region in 72.75%, 58.30%, 47.30%, 32.10%, and 29.50% of infested camels, respectively. Camel mange mite infection generally starts in the head region, extending through the neck to other areas with thin skin, such as the penile sheath and the udder. The whole body may become infested within a month.^28^ Richard also indicated that, camel mange infestation commences at areas of thin skin: the head, base of the neck, udder, prepuce and flank. The head becomes affected rapidly in every case because the animal uses its teeth to scratch the affected areas.^29^

This baseline results clearly indicated that camel mange mite infestation is present in the northern part of Ethiopia. Therefore, considering the zoonotic importance and effect of *Sarcoptes scabiei *var.* cameli *on camel production and productivity, more detailed investigation on the epidemiology, economic significance and species composition of this disease should be conducted to design and implement effective control programme, and improve the camel production and productivity.
